# System size reduction in stochastic simulations of the facilitated diffusion mechanism

**DOI:** 10.1186/1752-0509-6-121

**Published:** 2012-09-08

**Authors:** Nicolae Radu Zabet

**Affiliations:** 1 Cambridge Systems Biology Centre, University of Cambridge, Tennis Court Road, Cambridge CB2 1QR, UK; 2 Department of Genetics, University of Cambridge, Downing Street, Cambridge CB2 3EH, UK

**Keywords:** Simulation speed, Transcription factor copy number, DNA locus, Gene regulation, Transcription factor diffusion, Binding affinity, Occupancy profile, lacI

## Abstract

**Background:**

Site-specific Transcription Factors (TFs) are proteins that bind to specific sites on the DNA and control the activity of a target gene by enhancing or decreasing the rate at which the gene is transcribed by RNA polymerase. The process by which TF molecules locate their target sites is a key component of transcriptional regulation. Therefore it is essential to gain insight into the mechanisms by which TFs search for the target sites.

Research in this area uses experimental and analytical approaches, but also stochastic simulations of the search process. Previous work based on stochastic simulations focussed only on short sequences, primarily for reasons of technical feasibility. Many of these studies had to disregard possible biases introduced by reducing a genome-wide system to a smaller subsystem. In particular, we identified crucial parameters that require adjustment, which were not adequately changed in these previous studies.

**Results:**

We investigated several methods that adequately adapt the parameters of stochastic simulations of the facilitated diffusion, when the full sequence space is reduced to smaller regions of interest. We found two methods that scale the system accordingly: the *copy number model* and the *association rate model*. We systematically compared the results produced by simulations of the subsystem with respect to the original system. Our results confirmed that the *copy number model* is adequate only for high abundance TFs, while for low abundance TFs the *association rate model* is the only one that reproduces with high accuracy the results of the full system.

**Conclusions:**

We propose a strategy to reduce the size of the system that adequately adapts important parameters to capture the behaviour of the full system. This enables correct simulations of a smaller sequence space (which can be as small as 100 *Kbp*) and, thus, provides independence from computationally intensive genome-wide simulations of the facilitated diffusion mechanism.

## Background

Transcription Factors need to locate their target sites on the DNA within a time frame that is shorter than can be achieved by random diffusion. The search process is further complicated by the fact that target sites are usually similar to a significant number of other sites (decoys), and by the fact that there are other molecules searching for their target sites simultaneously. To understand transcriptional regulation better, it is therefore essential to have a complete understanding of the mechanistic way in which this search process takes place.

In the last 40 years, both theoretical and experimental research were able to identify that the search mechanism is a combination of a three-dimensional diffusion and a one-dimensional random walk, which is often referred to as the *facilitated diffusion* mechanism [1-6]. Despite considerable progress and mainly due to the technical limitations [7], there is still a significant gap in our understanding of how TFs locate their target sites [8]. One issue is the way in which the TF performs the one-dimensional random walk, in the sense that there is still no consensus whether the TF molecules predominantly slide (do not lose contact with the DNA during the one-dimensional random walk) [9-11] or hop (perform small jumps on the DNA during the one-dimensional random walk) [7,12]. Another example is the disagreement between the values for the proportion of time that TF molecules spend on the DNA: analytical computations of an optimal search process [13] differ from values measured experimentally [4].

One way to address these questions are stochastic simulations of the facilitated diffusion mechanism [14-16]. In [17,18] we proposed a computational model, GRiP, that allows genome-wide simulation of the facilitated diffusion mechanism. In particular, the CPU time required to simulate 1 *s* of an *E.coli* K-12 cell and lac repressor (lacI) TFs in GRiP resides between 1 *h* and 4 *h* on a 2×2.26 GHz quad-core Intel Xeon MacPro computer, see [17].

Despite the significant speed-up compared to previous tools, it is still not feasible to use the full genomic sequence as a search space. To address a scientific question with GRiP, multiple simulations need to be performed to allow a meaningful statistical analysis of the results. Thus, even small improvements in simulation speed can add up to some significant time saving. The optimization of the algorithm or of the implementation can potentially increase the speed of the simulations, but this is limited by the level of detail in the simulated model. In addition, even in the case of significant algorithm optimisations, simulating eukaryotic systems that have more than 100 *Mbp* and 10^7^ TFs becomes impractical.

One strategy to increase simulation speed consists of system size reduction, following the logic that the properties of the search process are the same irrespective of simulating only a subset or the full genomic sequence. However, this requires a few simulation parameters to be adapted to the size of the subsystem (e.g. the number of TF molecules in the subsystem as compared to the full system). This change in system parameters is required in order to avoid biases in the results, e.g. TFs could locate the target sites faster or target sites might be occupied for longer time intervals if there is an inappropriate number of TFs. The main advantage of this approach is that smaller systems will display faster speeds due to smaller DNA regions and, consequently, due to lower number of molecules bound to the DNA which perform the one-dimensional random walk.

Our results indicate that if the diffusion parameters are conserved and if the proportion of covered DNA is similar for the original system and the subsystem, then the subsystem captures the dynamic and steady state behaviour of the original system with negligible error.

In this contribution, we present two adaptation methods (the copy number method and the association rate method) that managed to keep the simulation results for the full system and the subsystem constant. We systematically investigate the degree to which the simulation results are affected when reducing the size of the system. The first method (*copy number method*) is simpler to implement, but is limited with respect to how much the system can be reduced and in terms of accuracy. This is caused by the fact that TF copy numbers are integers and values lower than 1 cannot be considered while intermediary values need to be rounded to the closest integer value.

The second approach, the (*association rate method*), is slightly more difficult to implement (it requires to measure the proportion of time the molecules spend on the DNA *a priori*), but surpasses all the limitations of the previous method (higher accuracy and the size of the smallest subsystem is not limited by TF copy number any more).

Overall, we show that copy number method performs well in the case of high abundance TFs, while for low abundance TFs, one needs to rely on the association rate method.

## Results and discussion

In this study we consider the lac repressor (lacI) TF, since this is one of the best described TFs with respect to the facilitated diffusion mechanism. Details regarding the lacI parameters used in this paper are presented in the Methods section. For the purpose of this study, we did not aim to provide a complete and exact description of the lac repressor system, but rather to describe under which conditions one can reduce the size of the system.

**Figure 1:**
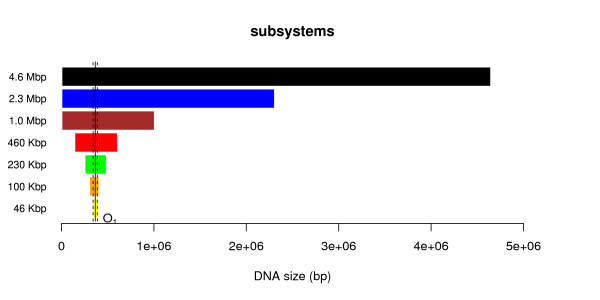
**DNA subsystems.** The vertical solid line indicates the position of the *O*_1_ site, while the vertical dashed line delimits the 46 *Kbp* region where we compare the occupancy bias correlation.

We consider six subsystems which are smaller than the full system (4.6 *Mbp*), namely: (*i*) 2.3 *Mbp*, (*ii*) 1.0 *Mbp*, (*iii*) 460 *Kbp*, (*iv*) 230 Kbp, (*v*) 100 *Kbp* and (*vi*) 46 *Kbp*. All subsystems contain the *O*_1_ site (which is the strongest site for the lac repressor in *E.coli*), see Figure 1. Note that, when reducing the system size, we keep the *O*_1_ site in the new DNA subsequence and, to avoid artefacts resulting from the boundary conditions, we keep the *O*_1_ site far from the DNA margins, see Figure 1.

**Figure 2:**
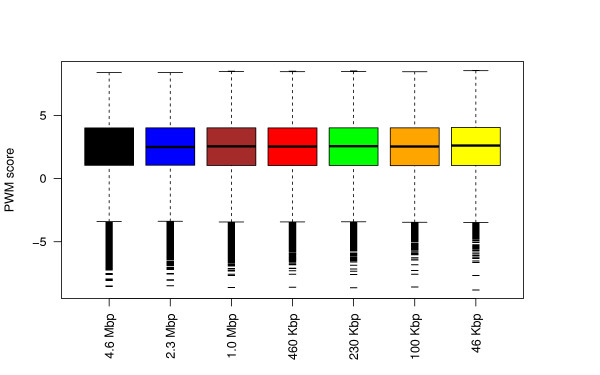
**Binding energy of lacI to all six subsystems.** In this graph we plotted the PWM score as box plot for the six subsystems and for the full system.

Figure 2 shows that the binding energy distribution of the six subsystems matches with high accuracy that of the full system. In addition, Figure 2 also shows that reducing the size of the system reduces the number of the lower outliers, and this might lead to mismatches between the results of the full system compared to the results of the subsystems.

Next we present two models that are intended to keep the subsystems equivalent to the full system with respect to the facilitated diffusion mechanism.

### Model I: TF copy number reduction

If the full system contains a DNA molecule of size *M*, base pairs and *TF* number of molecules, which are each bound *f * percent of the time to the DNA, then the expected number of molecules bound per base pair is

(1) $$\frac{\text{TF}\cdot f}{M}=\frac{TF^{\text{bound}}}{M}$$

where *TF*^bound ^represents the number of bound molecules.

A subsystem with a DNA molecule of size *λM* (with *λ* ∈[0,1]), is equivalent to the full system if the one-dimensional random walk behaviour in the two systems are the same and if the local crowding (the number of TF molecules bound to the DNA, normalised by the length of the DNA) remains the same in both the full system and the subsystem. We assume that the one-dimensional diffusion parameters are the same in all systems (which leads to similar one-dimensional diffusion measures, see *Additional file*1: *Supplementary Material*) and then we need to impose that the local crowding is also the same. One measure for crowding is the number of bound molecules per base pair and this leads to the following condition on crowding

(2) $$\frac{TF^{\text{bound}}}{M}=\frac{TF_{\lambda }^{\text{bound}}}{\lambda M}\Rightarrow TF_{\lambda }^{\text{bound}}=\lambda TF^{\text{bound}}$$

where $\left(TF_{\lambda }^{\text{bound}}\right)$ is the number of bound molecules in the subsystem *λ*.

One way to alter the number of bound molecules is to keep all the parameters the same and only reduce the total number of molecules in the cell proportionally to *λ*. This model (which we call the *copy number reduction model* or Model I) assumes that the TF copy number in the subsystem scales as:

(3) $$TF_{\lambda }=\lambda TF$$

### Model II: association rate reduction

Alternatively, the number of bound molecules can be modified without changing the total TF abundance, but only by changing the association rate of the molecules (*k*^assoc^) accordingly. In [18], we derive the association rate for the full system as

(4) $$k^{\text{assoc}}=\frac{1}{t_{R}}\frac{TF^{\text{bound}}}{TF^{\text{free}}}\cdot \frac{A_{i}^{\text{max}}}{A_{i}^{\text{total}}}$$

where *t*_*R*_ is the residence time of a molecule on the DNA, *TF*^free^ the number of free molecules and $\left(A_{i}^{\text{max}}/A_{i}^{\text{total}}\right)$ the ratio of free DNA. Note that, in the *Additional file*1: *Supplementary Material*, the accuracy of this estimate is systematically investigated in the case of DNA crowding. The results confirm that for a DNA occupancy up to 50% (as in the case of *E.coli*[19]) the equation displays negligible errors.

The ratio of bound TF is given by:

(5) $$\frac{TF^{\text{bound}}}{TF^{\text{free}}}=\frac{f\cdot \text{TF}}{(1-f)\cdot \text{TF}}=\frac{f}{1-f}$$

In the case of the subsystems, the association rate becomes

(6) $$k_{\lambda }^{\text{assoc}}=\frac{1}{t_{R}}\frac{TF_{\lambda }^{\text{bound}}}{TF_{\lambda }^{\text{free}}}\cdot \frac{A_{i}^{\text{max}}}{A_{i}^{\text{total}}}$$

In the association rate model, the total number of molecules in the system remains constant (only the number of molecules bound to the DNA decreases in the association rate model) and, thus, we have

(7) $$TF_{\lambda }^{\text{bound}}+TF_{\lambda }^{\text{free}}=TF^{\text{bound}}+TF^{\text{free}}$$

from equation (5):

(8) $$TF_{\lambda }^{\text{bound}}+TF_{\lambda }^{\text{free}}=TF^{\text{bound}}+TF^{\text{bound}}\frac{1-f}{f}$$

from equation (2):

(9) $$\begin{array}{lll} TF_{\lambda }^{\text{bound}} & +TF_{\lambda }^{\text{free}}=\frac{1}{\lambda }TF_{\lambda }^{\text{bound}}\frac{1}{f}\Rightarrow & \\ \frac{TF_{\lambda }^{\text{bound}}}{TF_{\lambda }^{\text{free}}} & =\frac{\text{fλ}}{1-\text{fλ}} \end{array}$$

The association rate of the subsystem ($\left(k_{\lambda }^{\text{assoc}}\right)$) scales by a term *γ* compared to the full system (*k*^assoc^).

(10) $$k_{\lambda }^{\text{assoc}}=\gamma k^{\text{assoc}}$$

from equations (5) and (9):

(11) $$\gamma =\frac{\lambda -\text{fλ}}{1-\text{fλ}}$$

### Comparison of the two models

**Figure 3:**
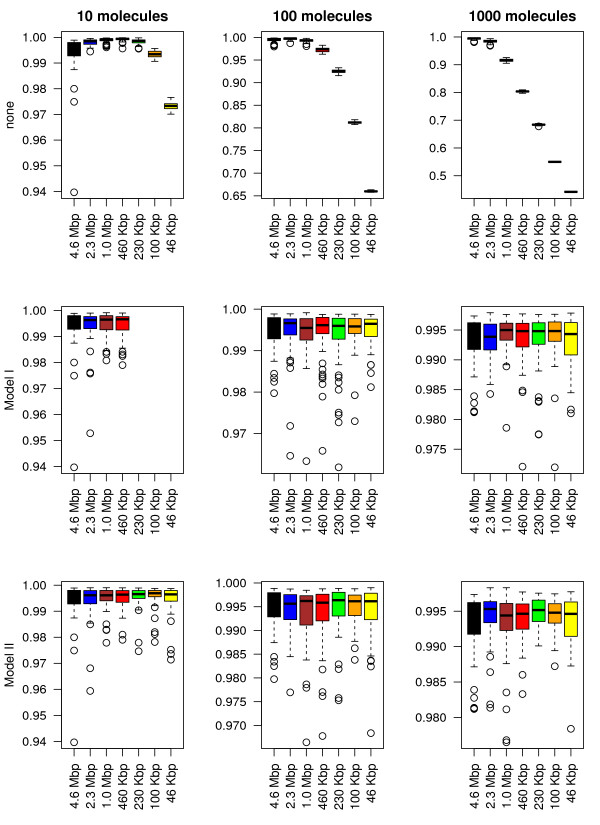
**Occupancy bias correlation between the full system and the subsystems.** We consider the smallest subsequence (46 Kbp) and the corresponding regions in all other sequences and we computed the Pearson correlation coefficient between occupancy biases. First, we compute the average occupancy bias for the full system using 60 independent simulations and then, for each simulation (including the full system), we compute the correlation of the current occupancy bias and the mean value of the full system. Only lacI molecules were added to the system and each simulation was run for: 2000 s (in the case of 10 molecules), 200 s (in the case of 100 molecules) and 20 s (in the case of 1000 molecules). On the first row none of the parameters are changed, while on the second and third ones, the number of lacI molecules and the association rate was varied according to the system size.

**Figure 4:**
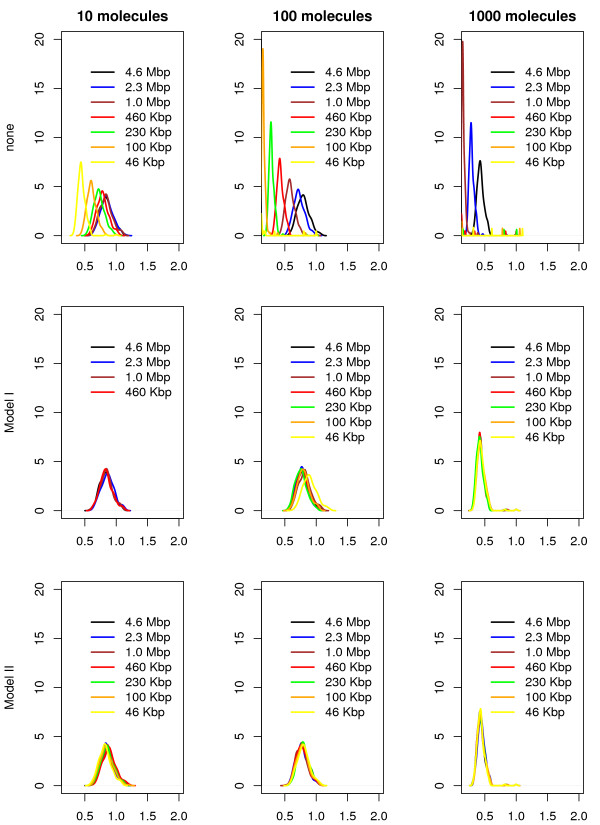
**The ratio between normalized affinity and normalized occupancy.** We consider the smallest subsequence (46 Kbp) and consider the top ≈ 180 sites (the binding energy is not lower than 30% compared to the strongest site). Only lacI molecules were added to the system and 60 simulations were run for each set of parameters, each simulation was run for: 2000 s (in the case of 10 molecules), 200 s (in the case of 100 molecules) and 20 s (in the case of 1000 molecules). On the first row none of the parameters are changed, while on the second and third ones the number of lacI molecules and the association rate was varied according to the system size.

**Figure 5:**
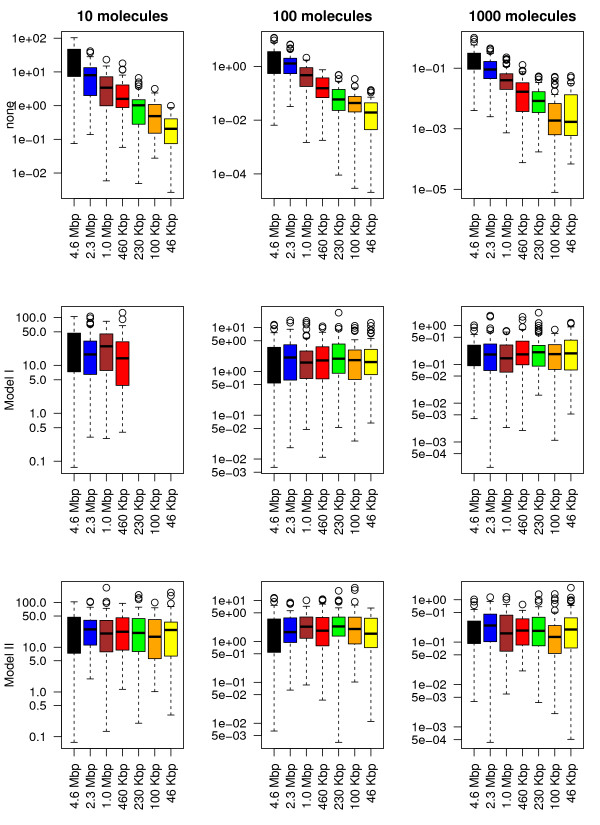
**Time to reach the target site.** 60 independent simulations were run, only lacI molecules were added to the system and each simulation was run for: 2000 s (in the case of 10 molecules), 200 s (in the case of 100 molecules) and 20 s (in the case of 1000 molecules). On the first row none of the parameters are changed, while on the second and third ones the number of lacI molecules and the association rate was varied according to the system size.

**Figure 6:**
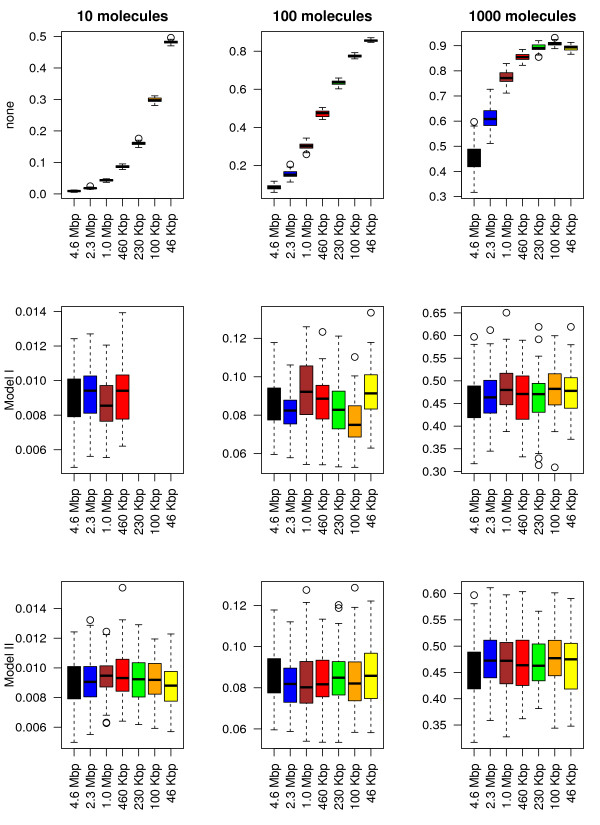
**The probability that the target site is occupied by a TF molecule.** The proportion of time the *O*_1_ target site was occupied by lacI molecules during the simulation time was measured using 60 independent simulations. Only lacI molecules were added to the system and each simulation was run for: 2000 s (in the case of 10 molecules), 200 s (in the case of 100 molecules) and 20 s (in the case of 1000 molecules). On the first row none of the parameters are changed, while on the second and third ones the number of lacI molecules and the association rate was varied according to the system size.

Next we will compare the two models (the copy number model and the association rate one) and investigate under which conditions one model is better than the other. The comparison includes four performance parameters, namely: (*i*) the occupancy bias, (*ii*) the time to first reach the target site, (*iii*) the probability that the target site is occupied and (*iv*) the simulation speed. Note that when none of the two methods are applied, the values of the first three properties in the subsystems will deviate significantly from the values of the full system; see top panels of Figures 3, 4, 5 and 6.

We consider three cases with respect to TF abundance, namely: (*a*) 1000 molecules (high abundance TFs), (*b*) 100 (medium abundance TFs) and (*c*) 10 (low abundance TFs). The corresponding values of the six subsystems for the abundances (for the copy number model, Model I) and for the association rates (for the association rate model, Model II) are listed in Table 1.

**Table 1:** Copy number and association rate for subsystems when we use the DNA size ratio method

**DNA size**		**lacI**			$k_{\lambda }^{\text{assoc}}s^{-1}$	
4.6 *Mbp*	1000	100	10		2400	
$\left(2.3\mathrm{Mbp}\right)$	496	50	5	172.28	169.09	168.75
1.0 *Mbp*	216	22	2	50.78	49.79	49.68
460 *Kbp*	99	10	1	20.60	20.19	20.15
230 *Kbp*	50	5	-	9.81	9.61	9.59
100 *Kbp*	22	2	-	4.15	4.07	4.06
46 *Kbp*	10	1	-	1.89	1.85	1.85

The full system consists of (*i*) 1000, (*ii*) 100 and (*iii*) 10 lacI molecules. When computing $\left(k_{\lambda }^{\text{assoc}}\right)$, we assumed that, for the full system, the time spent on the DNA is: *i*) 〈*f*_10_〉≈0.923, (*ii*) 〈*f*_100_〉≈0.922 and (*iii*) 〈*f*_1000_〉≈0.921.

Note that the association rate for the full system (2400 *s*^−1^) leads to a slightly different occupancy on the DNA than initially computed (*f*=0.9). This is due to the fact that the value of 2400 *s*^−1^was computed under the assumption that the system consists of both cognate and non-cognate molecules which cover 25% of the DNA. Since in this case we consider a significantly lower occupancy, then the proportion of time the TFs spend on the DNA increases. This is important due to the fact that the association rate model requires that the correct value for the proportion of time spent on the DNA (*f *) is provided.

The proportion of time spent on the DNA can be computed using the approach described in [18], but the accuracy can slightly suffer in the case of crowding on the DNA; see *Additional file*1: *Figure S4*. To ensure a parameter estimation characterised by a high accuracy, we used a set of 20 simulations and measured the observed proportion of time spent on the DNA (*f *); see *Additional file*1: *Figure S3*.

In addition, Table 1 shows that for low abundance proteins, it is not possible to apply Model I (copy number model) to reduce the size of the system beyond certain limits. For example, in the case of a low abundance protein with only 10 molecules per cell, the genome cannot be reduced further than 10 times (minimum size for 10 molecules is 460 *Kbp*).

#### Occupancy bias

Figure 3 shows that there is a strong correlation between the full system and the six subsystems in terms of occupancy biases. In particular, both models (copy number and association rate) seem to capture the same occupancy bias as the full system even for subsystems that are 100 times smaller, i.e., the boxplots of the correlations for all subsystems do not seem to deviate to much from that of the full system (leftmost). This is true for both models (copy number and association rate model) and seem to be valid for all types of TF abundances (low, medium or high). Nevertheless, because TF copy numbers are integers, applying large system reduction to low abundance proteins can result in a lower accuracy of the method.

The correlation between the occupancy bias of the full system and all the subsystems indicates that the peaks in the occupancy bias data are captured by all subsystems for both models (copy number and association rate models). However, to capture the complete perspective on the occupancy bias we need to investigate if the size of these peaks is conserved, i.e., we are interested whether the same ratio between occupancy and affinity is found in the subsystems as compared to the full system. To do this, we use the ratio between normalized affinity and normalized occupancy for all sites that have a certain minimum affinity. This minimum affinity threshold removes low affinity sites from the data, where the noise in occupancy bias is high (and could lead to misinterpretation of the data).

For the sites with the affinity above a certain value we compute the ratio between the normalized affinity and the normalized occupancy. In the low and medium abundance TFs we expect the ratio to be around one, but in the case of high abundance TFs, due to the high crowding, the ratio should be significantly lower than 1 (resulting in many false positives, in the sense that these sites are identified as highly occupied sites, with prospective high affinity, but the actual affinity is lower than predicted based the occupancy) [18,20].

Figure 4 shows that in both models the size of the system can be reduced while displaying similar distributions of the ratio between affinity and occupancy. Interestingly, in a highly crowded environment, there seems to be a high peak centred around 0.5 (higher occupancy than affinity; false positives), but also a small number of values around 1 (similar degree of affinity to occupancy). However, we found that, for low/medium abundance TFs, the copy number model in conjunction with the smallest subsystem (46 *Kbp*) can lead to results that deviate significantly from the ones of the full system (the top second panel in Figure 4). Overall, we conclude that the association rate model always performs well and that for high abundance proteins, both models show good results.

#### Time to reach the target site

Next, we are interested in how the system size reduction influences the search process. Figure 5 shows that, for both models, reducing the size of the system does not significantly change the time required to locate the target site for all types of TF abundances. Again, the association rate model seems to slightly outperform the copy number model, due to higher accuracy of how the scaling factor is incorporated into the model, i.e., in Model I the copy number can take only integers, resulting in lower accuracy for low abundance TFs, while for Model II due to the fact the association rate can take real positive numbers the accuracy is only limited by the accuracy of the floating point number representation in computers.

#### The probability that the target site is occupied

Usually, the activity of TF regulated genes is controlled by the presence or absence of TF molecules at certain target sites. Using our model, we measured the proportion of time the target site was occupied. For long time intervals, this proportion of time approximates the probability that a target site is occupied by a TF.

Figure 6 calculates the probability that the target site is occupied in the full system compared to the one of the subsystems for both models. Both models seem to approximate the behaviour of the full system with negligible error. In particular, both the average value and the variability in the probabilities seem to be conserved in all subsystems.

#### Simulation speed

**Figure 7:**
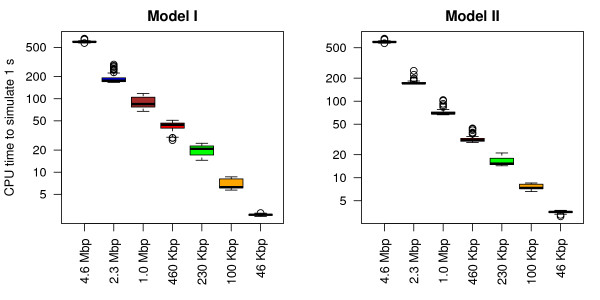
**Time required to simulate *1 s *in the cell for 1000 molecules.** The time required to simulate 1 s in the cell was measured on a Mac Pro 2 × 2.26GHz quad-core Intel Xeon with 32GB memory running Mac OSX 10.6.8. We produced 60 independent simulations for each set of parameters. In addition, each simulation was run for 20 s and only lacI molecules were added to the system. The number of lacI molecules and the association rate was varied according to the system size.

The main reason we reduced the size of the system was to increase simulation speed. Figure 7 shows that one can obtain a significant decrease in the CPU time required to simulate 1 s by decreasing the size of the system being simulated. Both models performed similarly. This speed-up is not only a consequence of having a fewer number of molecules performing the one-dimensional random walk, but also of a significant increase in the number of events processed per second. For example the machine used simulates 1 s of the full system in approximately 500 s, but a 100 times smaller system (46 Kbp) in less than 5 s.

Both models produce accurate results compared to the full system and lead to significant enhancement of the simulation speed. In particular, the errors in the approximate subsystems compared to the full system are negligible and are overshadowed by the speed enhancement produced by these methods.

## Conclusions

When simulating the facilitated diffusion mechanism, one usually needs multiple long runs for the same set of parameters. This can take a significant amount of CPU time and can lead to undesirable simulation time (greater than 2 months). One solution is to enhance the current algorithms, but this might lead to coarser grained models unable to capture enough details of the mechanism of facilitated diffusion. Alternatively, one could simulate a subsystem of the full system. Figure 7 shows that by decreasing the system size 100 times the CPU time required to simulate 1 *s* in the cell can be decreased 100 fold.

To keep the full system and the subsystems equivalent, we developed two models: (*i*) the copy number model (Model I) and (*ii*) the association rate model (Model II). Model I is easier to construct, but has two main drawbacks. First, there is a limit on how much one can reduce the system due to the fact that TF copy number has to be at least 1. This is mainly an issue for low abundance proteins (e.g. for a TF with 10 molecules, the smallest system one could obtain is 460 *Kbp*). Secondly, due to the fact that TF copy numbers are integers the accuracy of the method might suffer. For example, when we reduce the size of the system from 4.6 *Mbp*to 46 *Kbp* for a TF with 100 molecules the ratio between the affinity and the occupancy of the subsystem deviates significantly from that of the full system. Nevertheless, this approach is easy to apply and displays negligible errors for high abundance proteins.

The association rate model surpasses both drawbacks of the copy number model by managing to reduce the system independently of TF copy number and reproduces the results of the full system with high accuracy. However, this model assumes measuring the actual time the TF molecules spend on DNA in the full system *a priori*, which might be time consuming.

In the context of GRiP software [17,18] this indicates that, when we reduce the system size of high abundance TFs (e.g. non-cognate TF have ∼10^5^molecules), one could use the copy number model, while, for low abundance proteins, the association rate model needs to be applied.

In conclusion, this paper offers a comprehensive description and analysis of the methods that need to be applied when performing non-genome-wide stochastic simulations of the facilitated diffusion mechanism. More specifically, we show that one does not have to perform genome-wide studies of the TF search process for their target sites as long as the parameters of subsystem (the subsystem which considers only a small area around the region of interest) are correctly adjusted.

## Methods

### Lac repressor

We consider the case of the lac repressor in *E.coli* K-12 [21]. The lac repressor tetramer has only three known high affinity sites [22]: (*i*) AATTGTGAGCGGATAACAATT, (*ii*) AAATGTGAGCGAGTAACAACC and (*iii*) GGCAGTGAGCGCAACGCAATT. To construct the PWM of the lac repressor we use these three sites, but we assume that there is a 9 *bp* gap in the middle of the motif, which leads to the following three sequences: (*i*) AATTGTNNNNNNNNNACAATT, (*ii*) AAATGTNNNNNNNNNACAACC and (*iii*) GGCAGTNNNNNNNNNGCAATT. This assumption is justified by the fact that the lac repressor tetramer consists of two dimers, each recognising only 6 *bp*[23].

To compute the PWM we use the information theory based approach proposed in [24] (see [18]).

(12) $$E_{\text{lacI}}^{j}=\overset{L}{\underset{k=0}{\sum }}\ln\left(\frac{\nu_{j,k}^{\text{lacI}}}{\nu_{\left(j+k\right)}}\right)$$

where *L* is the length of the motif, *j* is the position on the DNA where we compute the binding energy and *k* is the position in the motif. If at position (*j* + *k*) on the DNA we have nucleotide *x*, then the frequency of this nucleotide at position *k* in all known high affinity binding sites is denoted by *ν*_*j*,*k*_ and its frequency in the entire genome by *ν*_(*j* + *k*)_. To ensure that the frequency in the motif is non zero we insert a pseudo-count term *ζ* when computing the frequency in the PFM [25].

(13) $$\nu_{j,k}^{\text{lacI}}=\frac{n_{j,k}^{\text{lacI}}+\zeta \cdot \nu_{j}}{\sum_{x\in \{A,C,G,T\}}n_{x,k}^{\text{lacI}}+\zeta }$$

**Figure 8:**
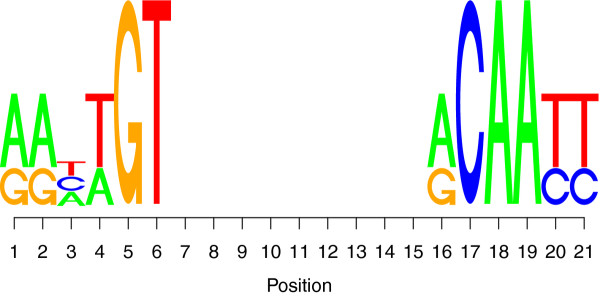
LacI sequence logo.

Using a pseudo-count value of *ζ*=1 and the nucleotide frequencies in *E.coli* K-12 [21], *ν*_*A*_=0.246, *ν*_*C*_=0.254, *ν*_*G*_=0.254, *ν*_*T*_=0.246 [26], we obtained the sequence logo shown in Figure 8. Note that the PWM matrix can be found in the *Additional file*1: *Supplementary Material*.

The binding energies for the entire *E.coli* K-12 genome [21] are normally distributed with mean 〈*E*_lacI_〉/*K*_*B*_*T*=2.47 and standard deviation of 2.16. In addition, the mean of the exponential binding energy is 〈exp (*E*_lacI_)〉=1.04. Using the approach described in [18], we computed the specific waiting time $\left(\tau_{\text{lacI}}^{0}=1.18e-06\right)$. Furthermore, we assume that the average length of the binding motif in prokaryotes is 23 *bp*[27], the average DNA occupancy is 0.25∈[0.1,0.5][19], the system has 50000 non cognate TFs and an association rate of *k*^assoc^=2400 *s*^−1^. The rest of the parameters describing this system are detailed in [18]. The selected parameters resulted in an average time spent on the DNA of *f*≈0.88, a residence time of *t*_*R*_≈4.5 *ms* and a sliding length of $\left(s_{l}^{\text{obs}}\approx 87\mathrm{bp}\right)$, which are in accordance with the values estimated in [4].

The lac repressor has three sites that control the activity of the lac operon, namely: *O*_1_, *O*_2_ and *O*_3_(see *Additional file*1: *Supplementary Material*). Our PWM matrix correctly predicts that *O*_1_ is the strongest site on the DNA and, in our analysis, we will use this site when we measure the time required for a lacI molecule to reach a target site or when we measure the proportion of time the target site was occupied by lacI molecules.

## Competing interests

The author declares that he has no competing interests.

## Authors’ contributions

NRZ designed the study, performed the analysis and wrote the paper.

## Supplementary Material

Additional file 1 **Supplementary Material. **The *Supplementary Material* contains extra figures which confirm that the measured one-dimensional parameters are conserved in the subsystems. These parameters include: residence time, sliding length, actual sliding length and proportion of time the molecules spend on the DNA. In addition, the *Supplementary Material* contains an alternative method to scale the system which assumes that λ (the scaling factor) is determined by the ratio between sum of all waiting times in the subsystem and sum of all waiting times in the full system. Click here for file
