# Supplementary material for: System size reduction in stochastic simulations of the facilitated diffusion mechanism
Source: BMC Syst Biol. 2012 Sep 8;6:121. doi: 10.1186/1752-0509-6-121 (PMC3567987; doi:10.1186/1752-0509-6-121)
Supplement: Additional file 1 — Supplementary Material. The Supplementary Material contains extra figures which confirm that the measured one-dimensional parameters are conserved in the subsystems. These parameters include: residence time, sliding length, actual sliding length and proportion of time the molecules spend on the DNA. In addition, the Supplementary Material contains an alternative method to scale the system which assumes that λ (the scaling factor) is determined by the ratio between sum of all waiting times in the subsystem and sum of all waiting times in the full system. [file 1752-0509-6-121-S1.pdf]

Supplementary Material to: System size reduction in stochastic simulations of the facilitated diffusion mechanism.

Nicolae Radu Zabet[1,2,*]

[1]Cambridge Systems Biology Centre, University of Cambridge, Tennis Court Road, Cambridge CB2 1QR, UK

[2]Department of Genetics, University of Cambridge, Downing Street, Cambridge CB2 3EH, UK

[*]Corresponding author: n.r.zabet@gen.cam.ac.uk

1 Lac repressor

The PFM of the lacI is presented in Table 1.

The binding energy of lacI and the *E.coli* K-12 genome follows a normal distribution, see Figure 1.

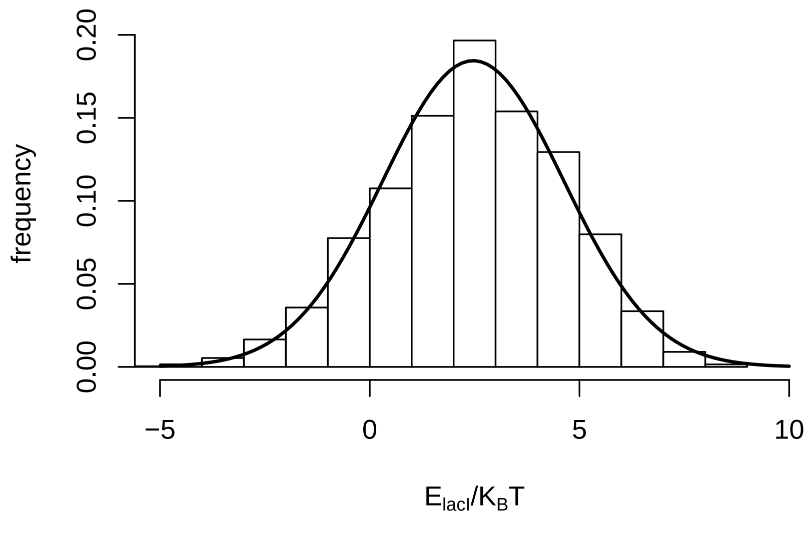

Figure 1: *lacI binding energy histogram* lacI binding energy histogram. We considered the genome of *E.coli* K-12 (Riley et al., 2006) and the computed PWM in conjunction with the Stormo (2000) algorithm.

The lac repressor has three sites that control the activity of the lac operon, namely: O_1, O_2 and O_3 (see Table 2). The order of the PWM score is conserved as previously estimated, (Vilar, 2010), but the third operator has a significantly low score.

	**PFM**				**PWM**			
**Position**	A	C	G	T	A	C	G	T
1	0.67	0.00	0.33	0.00	0.6200	−0.6900	0.1400	−0.6900
2	0.67	0.00	0.33	0.00	0.6200	−0.6900	0.1400	−0.6900
3	0.33	0.33	0.00	0.34	0.1600	0.1400	−0.6900	0.1800
4	0.33	0.00	0.00	0.67	0.1600	−0.6900	−0.6900	0.6200
5	0.00	0.00	1.00	0.00	−0.7000	−0.7000	0.9000	−0.7000
6	0.00	0.00	0.00	1.00	−0.6900	−0.6900	−0.6900	0.9300
7	0.25	0.25	0.25	0.25	0.0077	−0.0084	−0.0073	0.0083
8	0.25	0.25	0.25	0.25	0.0077	−0.0084	−0.0073	0.0083
9	0.25	0.25	0.25	0.25	0.0077	−0.0084	−0.0073	0.0083
10	0.25	0.25	0.25	0.25	0.0077	−0.0084	−0.0073	0.0083
11	0.25	0.25	0.25	0.25	0.0077	−0.0084	−0.0073	0.0083
12	0.25	0.25	0.25	0.25	0.0077	−0.0084	−0.0073	0.0083
13	0.25	0.25	0.25	0.25	0.0077	−0.0084	−0.0073	0.0083
14	0.25	0.25	0.25	0.25	0.0077	−0.0084	−0.0073	0.0083
15	0.25	0.25	0.25	0.25	0.0077	−0.0084	−0.0073	0.0083
16	0.67	0.00	0.33	0.00	0.6200	−0.6900	0.1400	−0.6900
17	0.00	1.00	0.00	0.00	−0.7000	0.9000	−0.7000	−0.7000
18	1.00	0.00	0.00	0.00	0.9300	−0.6900	−0.6900	−0.6900
19	1.00	0.00	0.00	0.00	0.9300	−0.6900	−0.6900	−0.6900
20	0.00	0.33	0.00	0.67	−0.6900	0.1400	−0.6900	0.6200
21	0.00	0.33	0.00	0.67	−0.6900	0.1400	−0.6900	0.6200

Table 1: lacI PFM and PWM

**site**	**left position**	**right position**	**PWM**
O_1	365547	365567	−8.49
O_2	365146	365166	−7.50
O_3	365639	365639	−2.64

Table 2: lacI target sites

DNA size	left position	right position
4.6 Mbp	0	4639675
2.3 Mbp	0	2300000
1.0 Mbp	0	1000000
460 Kbp	140000	600000
230 Kbp	250000	480000
100 Kbp	300000	400000
46 Kbp	342000	388000

Table 3: DNA subsystems

2 DNA subsystems

In Table, 3 we present six subsystems that have shorter DNA strands.

Figure 2 shows the binding energy for all six subsystems and confirms that the binding energy distribution does not deviate significantly from that of the full system.

3 Bound time to the DNA

The time the TF spends on the DNA for the three TF abundance cases (10, 100 and 1000 molecules) differs from $f = 0.9$ due to the fact that, when the association rate was computed, we assumed that 25% of the DNA is occupied, which is not the case any more. Figure 3 shows that the proportion of time the TF is bound to the DNA differs slightly from the target value $f = 0.9$, and this is important when computing the association rate.

$$\begin{aligned} \langle f_{10} \rangle &= 0.923064488 \\ \langle f_{100} \rangle &= 0.922896708 \\ \langle f_{1000} \rangle &= 0.921332824 \end{aligned} \tag{1}$$

4 Association rate accuracy

The estimate for the association rate equation derived in Zabet and Adryan (2012) is prone to reduced accuracy for high crowding on the DNA. In Figure 4 we systematically investigate the accuracy of the estimated one-dimensional statistics when the association rate is set as proposed in Zabet and Adryan (2012). The results confirm that for a DNA coverage of at least up to 50%, the difference between the estimated values and those observed in the simulations are negligible. Hence, the association rate estimation equation can be used further with high confidence.

5 One dimensional diffusion statistics

Figures 5, 6, 7 and 8 show that the statistics related to the one dimensional random walk (residence time, sliding length, actual sliding length and time bound to the DNA) from the subsystems do not deviate significantly from those in the full system. Note that the smaller

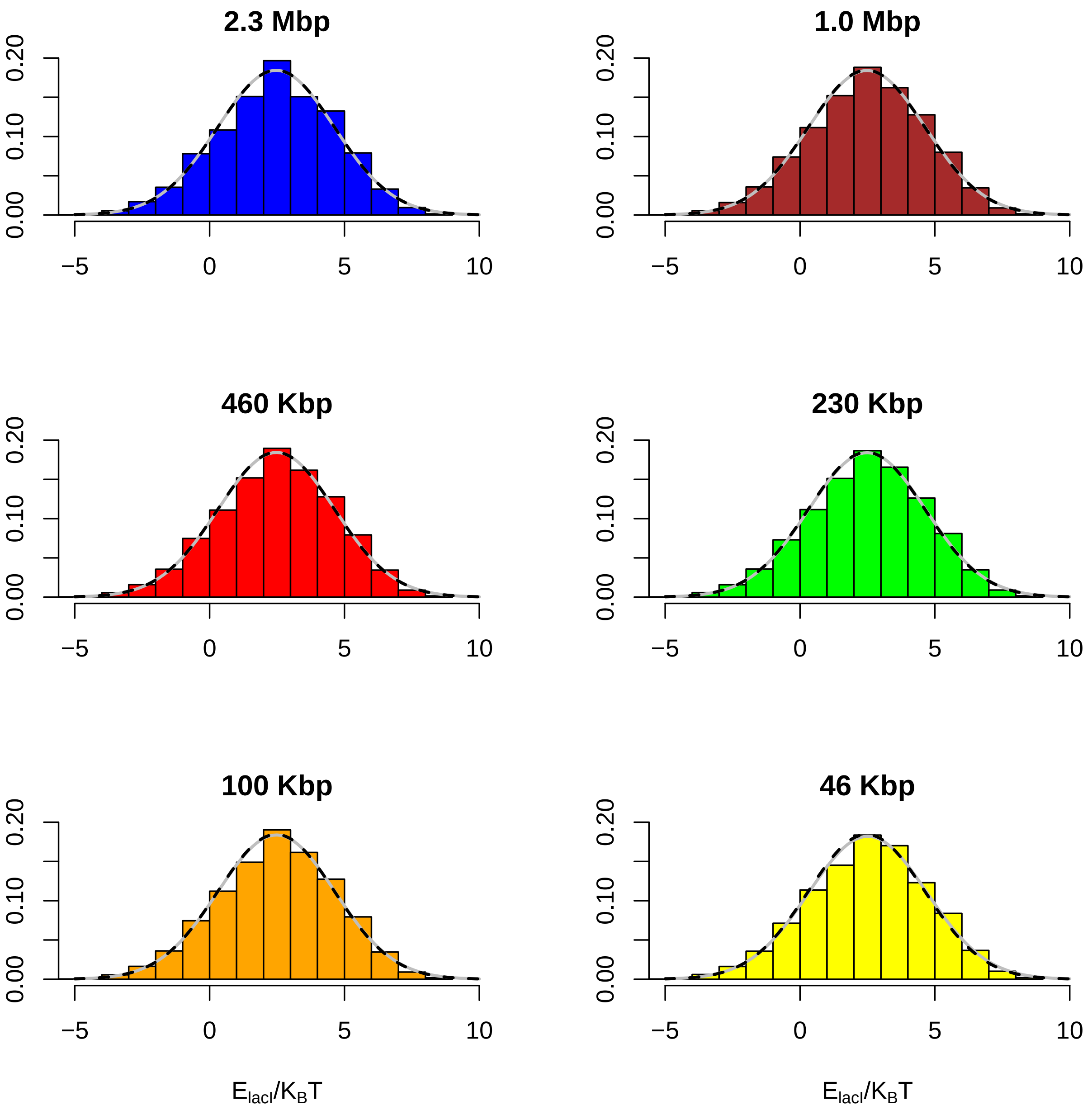

Figure 2: *lacI binding energy histogram for all six subsystems* The black dashed line represents the binding energy histogram for the full system. We plotted histograms for the six subsystems. The binding energies of the subsystems capture the details of the complete DNA sequence with high accuracy.

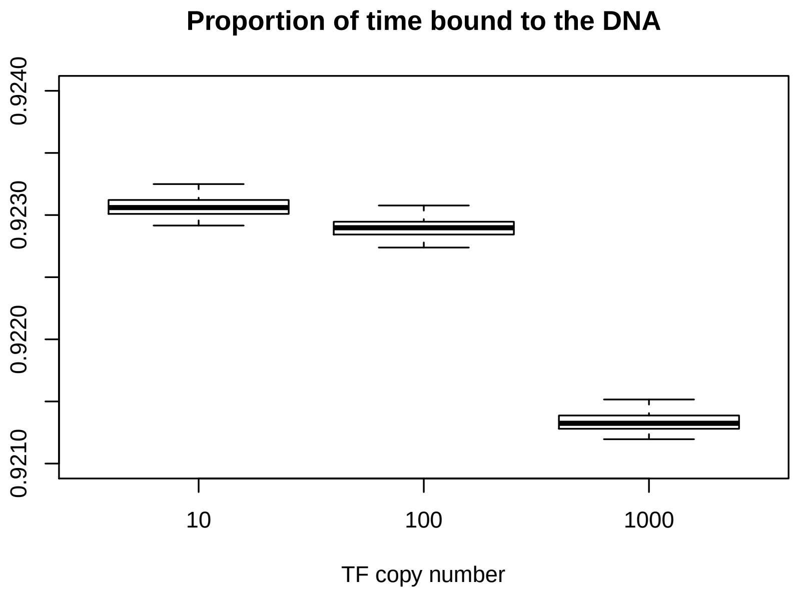

Figure 3: *The proportion of the time a TF molecule is bound to the DNA*. This value is for the full system and we considered three cases with respect with TF copy number: (i) 10, 100 and 1000 molecules.

subsystems seem to display slightly more variation with respect to these random walk parameters compared to the bigger system.

6 Total affinity scale factor

A different strategy to scale the subsystems assumes that λ represents the ratio between the sum of all waiting times in the full system and the sum of all waiting times in the subsystem.

$$\lambda = \frac{\sum_{j=pos_{\text{start}}}^{pos_{\text{end}}} \tau_j}{\sum_{j=1}^{M} \tau_j} \tag{2}$$

where pos_{start} is the absolute start position on the DNA of the subsystem and pos_{end} the absolute end position.

When using the total affinity as the scale factor we obtained the values for the copy numbers and association rates listed in Table 4.

Figure 9 shows that for large subsystems the scaling factor computed using the DNA size and the one computed using the total affinity have similar values. However, for low values, there seem to be some differences between the two approaches to compute the scaling factor.

Note that, when we used this approach to compute the association rate or TF copy numbers for the two models, we obtained similar results as the DNA size approach for occupancy bias, time to reach the target site and one dimensional statistics, see Figures 10, 12, 14, 15, 16 and 17. However, it seems that this approach to compute the scaling factor leads to an increase in the proportion of time the target site is occupied when the system size is reduced, see Figure 13.

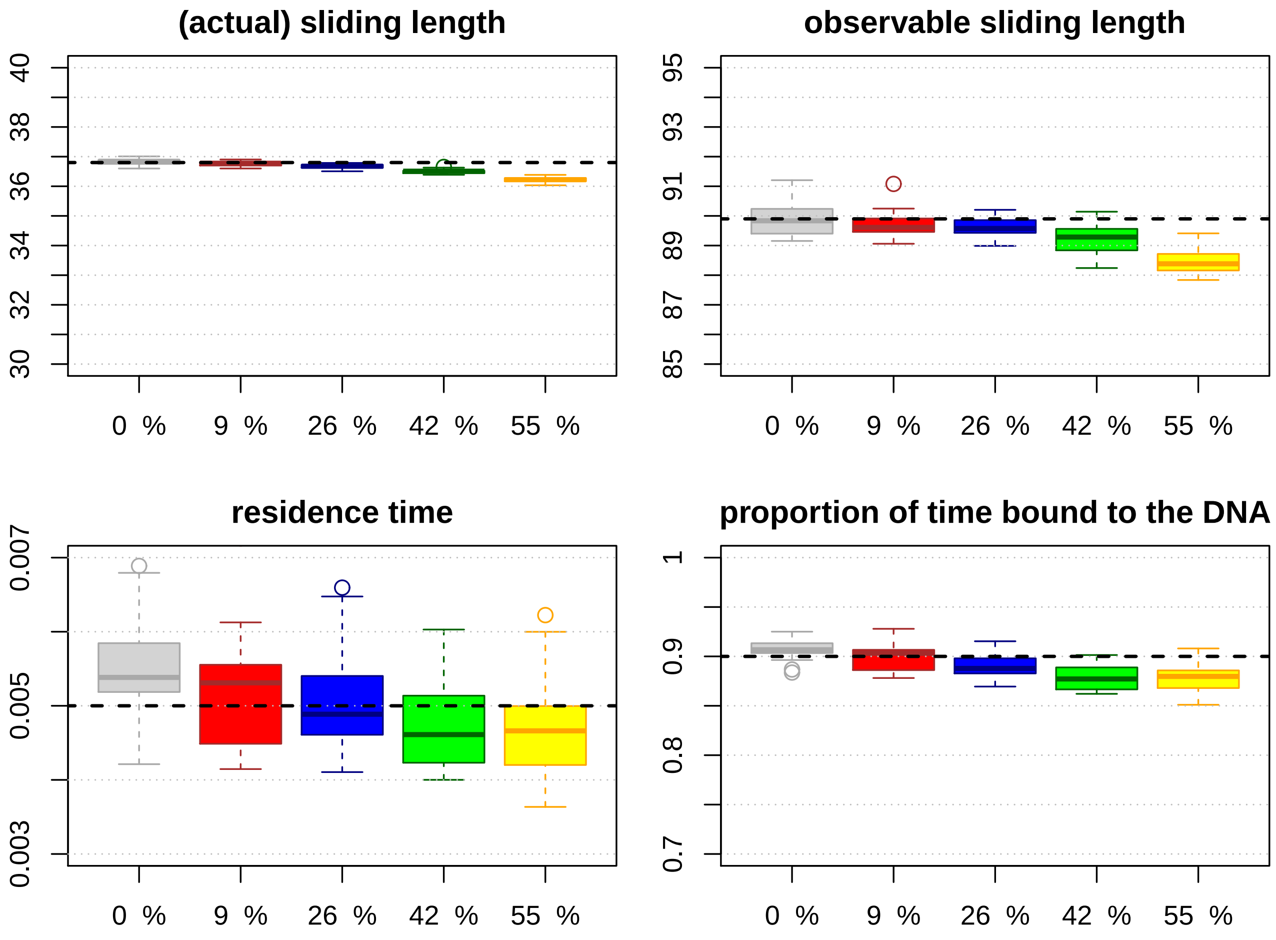

Figure 4: *1D statistics in a crowded environment.* We ran 20 independent simulations where lacI molecules and non-cognate molecules were added to the system and each simulation was run for 10 s. For the lacI TF we used the Gerland et al. (2002) algorithm and the following sequence motif: AATTGTNNNNNNNNNNACAATT. We considered 5 lacI molecules and the abundance of non-cognate TF was varied as follows: (i) 0, (ii) 10^4, (iii) 3×10^4, (iv) 5×10^4 and (v) 7×10^4. We used the association rate estimate equation derived in Zabet and Adryan (2012) and this led to the following association rates for both lacI and non-cognate TFs: (i) 1800 s^{-1}, (ii) 2000 s^{-1}, (iii) 2571 s^{-1}, (iv) 3600 s^{-1} and (v) 6000 s^{-1}. These values for the association rates led to values specified on the x-axis as the percentage of DNA being covered. Each non-cognate TF covered 46 bp. The horizontal dashed lines (black) correspond to the computed values.

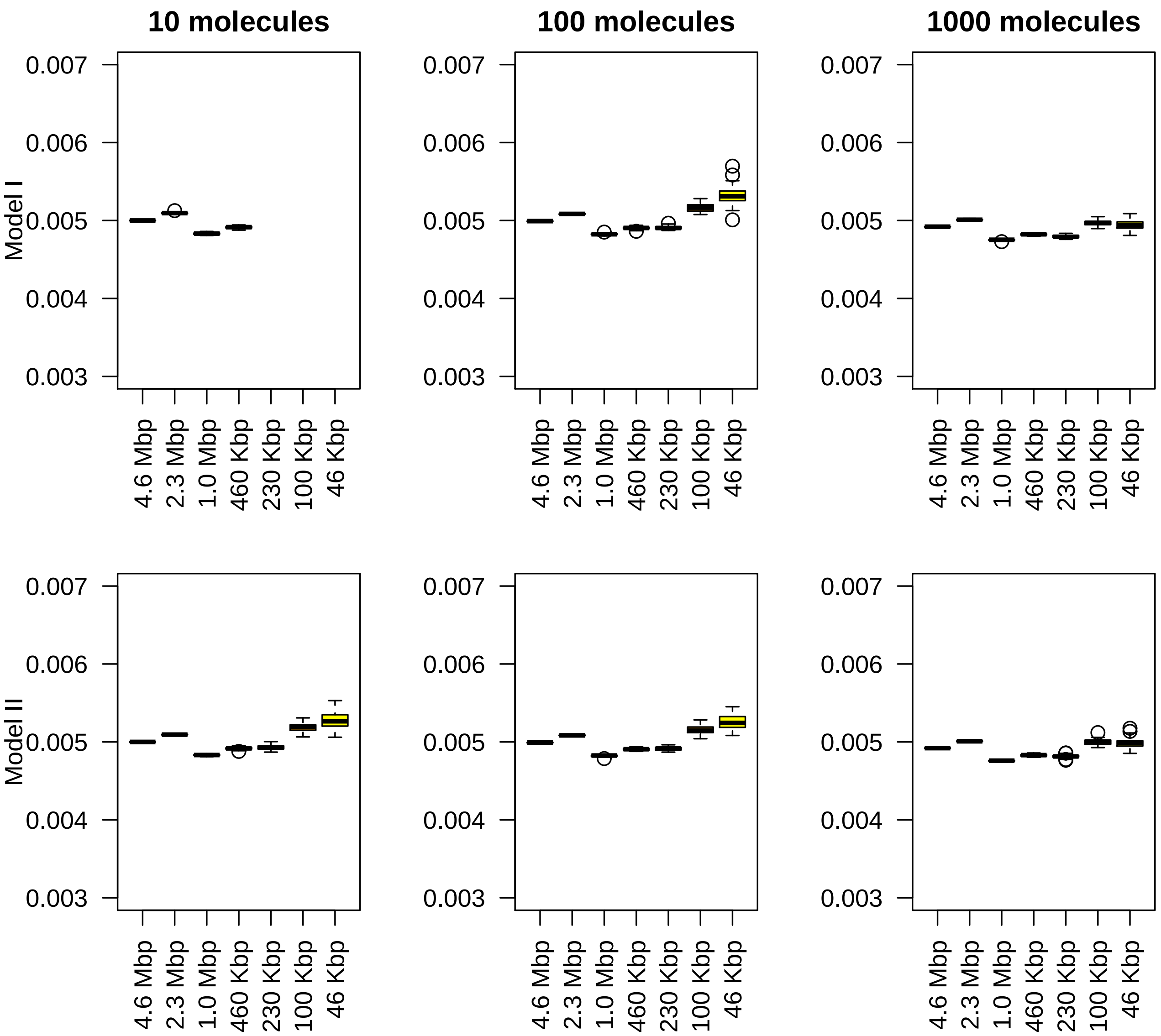

Figure 5: *Residence time per binding.* We ran 60 independent simulations, only lacI molecules were added to the system and each simulation was run for: 2000 s (in the case of 10 molecules), 200 s (in the case of 100 molecules) and 20 s (in the case of 1000 molecules). The number of lacI molecules and the association rates were varied according to the system size.

**DNA size**	**lacI**			$k_{\lambda}^{\text{assoc}}$ s^{-1}		
4.6 Mbp	1000	100	10	2400		
2.3 Mbp	500	50	5	174.96	171.73	171.38
1.0 Mbp	216	22	2	50.77	49.78	49.67
460 Kbp	100	10	1	20.81	20.40	20.35
230 Kbp	52	5	-	10.22	10.01	9.99
100 Kbp	23	2	-	4.45	4.36	4.35
46 Kbp	12	1	-	2.29	2.25	2.24

Table 4: *Copy number and association rate for subsystems when we use the total affinity ratio.* The full system consists of (i) 1000, (ii) 100 and (iii) 10 lacI molecules. When computing $k_{\lambda}^{\text{assoc}}$, we assumed that, for the full system, the time spent on the DNA is: (i) $\langle f_{10} \rangle \approx 0.923$, (ii) $\langle f_{100} \rangle \approx 0.922$ and (iii) $\langle f_{1000} \rangle \approx 0.921$.

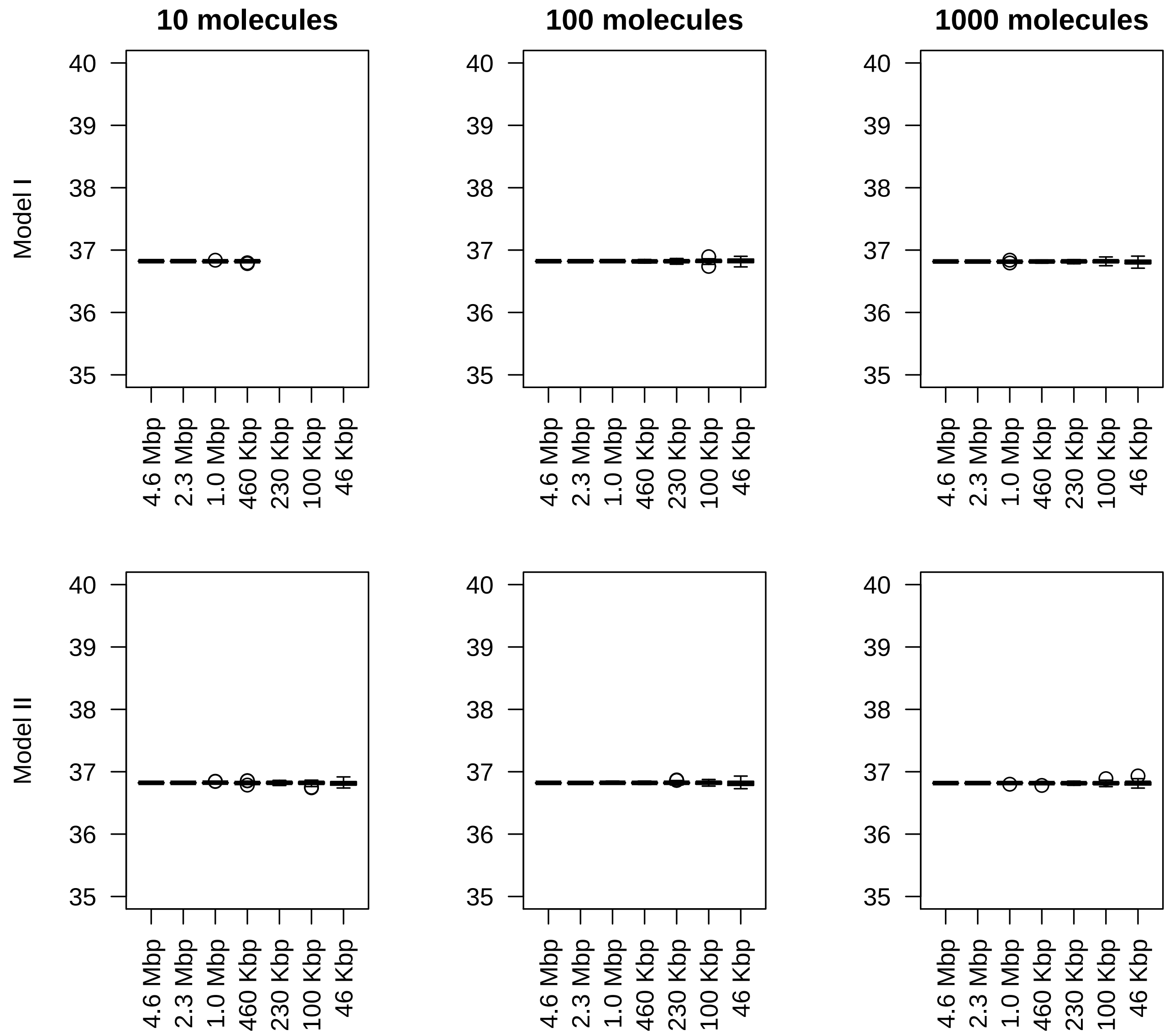

Figure 6: *Sliding length.* We ran 60 independent simulations, only lacI molecules were added to the system and each simulation was run for: 2000 s (in the case of 10 molecules), 200 s (in the case of 100 molecules) and 20 s (in the case of 1000 molecules). The number of lacI molecules and the association rates were varied according to the system size.

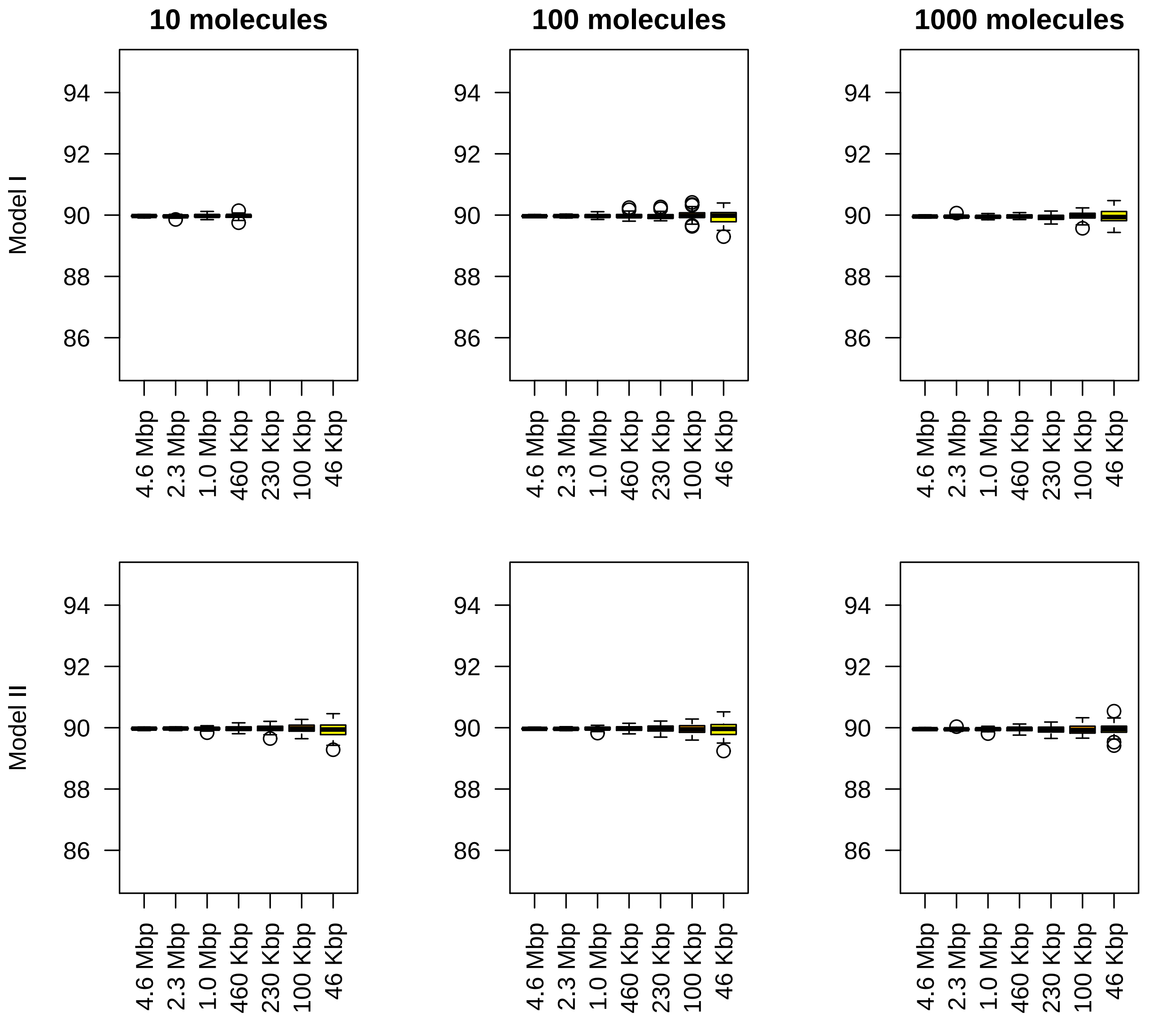

Figure 7: *Actual sliding length.* We ran 60 independent simulations, only lacI molecules were added to the system and each simulation was run for: 2000 s (in the case of 10 molecules), 200 s (in the case of 100 molecules) and 20 s (in the case of 1000 molecules). The number of lacI molecules and the association rates were varied according to the system size.

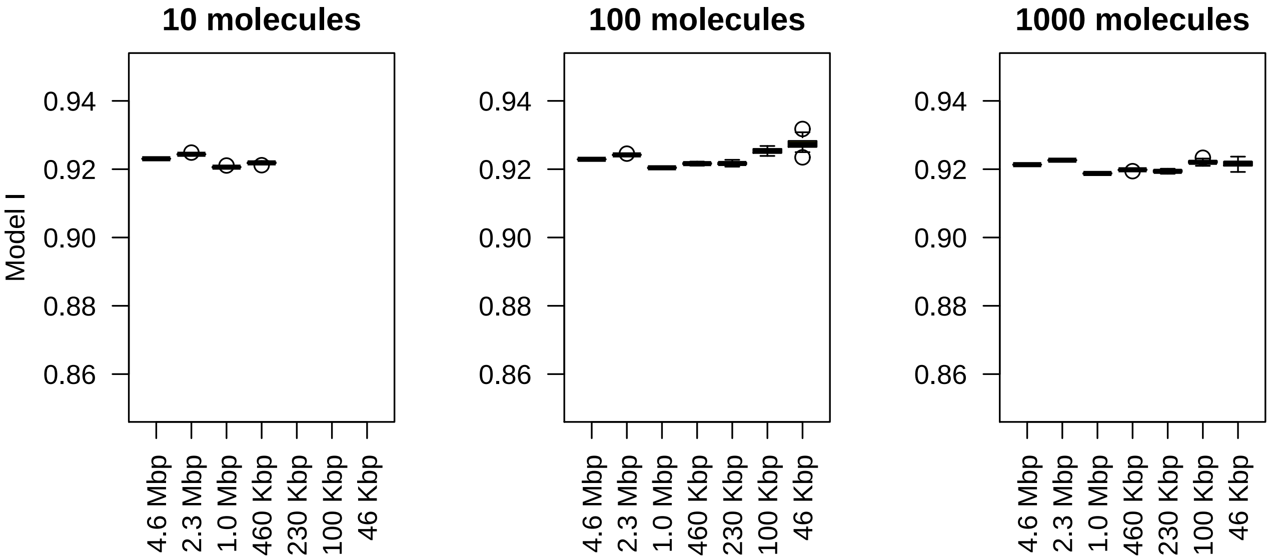

Figure 8: *The proportion of time a TF molecule is bound to the DNA.* We ran 60 independent simulations, only lacI molecules were added to the system and each simulation was run for: 2000 s (in the case of 10 molecules), 200 s (in the case of 100 molecules) and 20 s (in the case of 1000 molecules). The number of lacI molecules and the association rates were varied according to the system size. The values for Model II decrease due to the fact that we alter the parameters in order to obtain less time spent on the DNA.

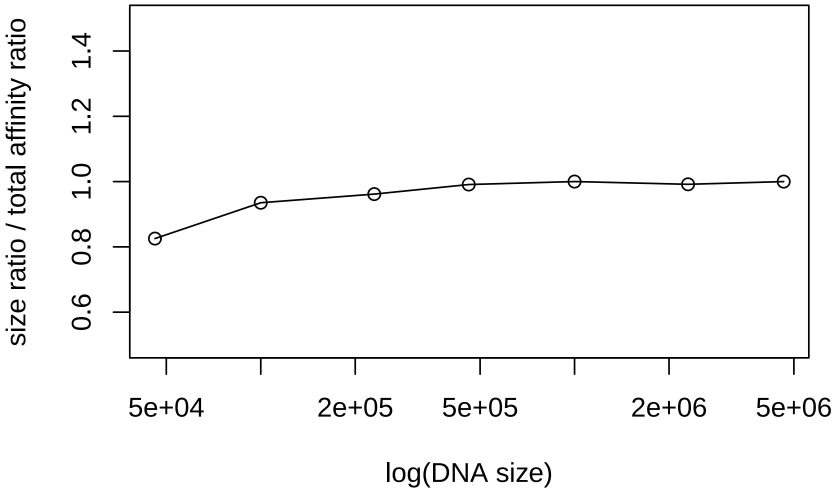

Figure 9: *Comparison between total affinity scale factor and DNA size one.* A value of 1 indicates that the two methods to compute the scaling factor (total affinity or DNA size) produce similar results.

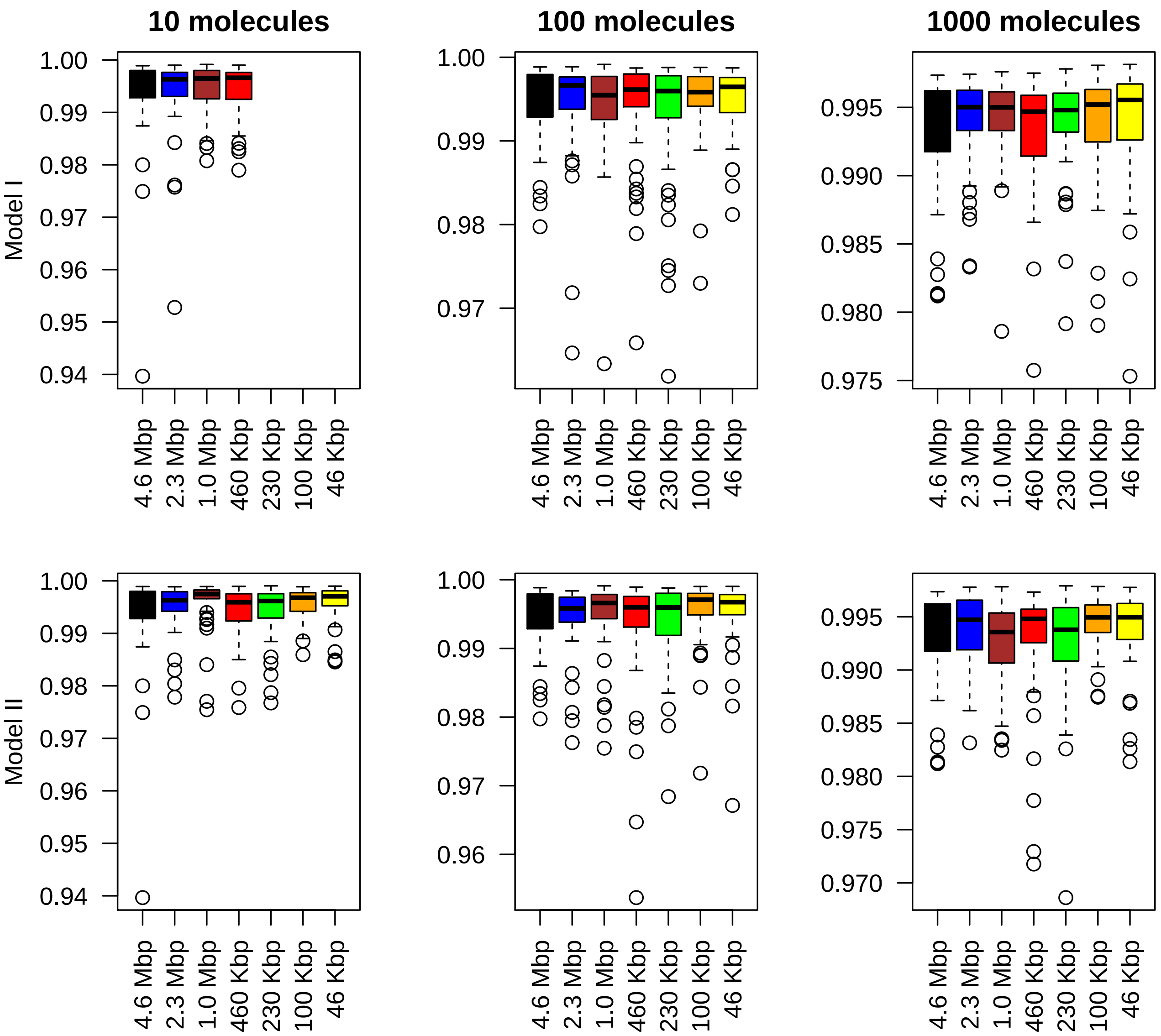

Figure 10: *The occupancy bias correlation between the full system and the subsystems when the subsystems are scaled by the total affinity ratio* We consider the smallest subsequence (46 Kbp) and the corresponding regions in all other sequences and we compute the Pearson coefficient of correlation between occupancy biases. First, we compute the average occupancy bias for the full system using 60 independent simulations and then, for each simulation (including the full system), we compute the correlation of the current occupancy bias and the mean value of the full system. Only lacI molecules were added to the system and each simulation was run for: 2000 s (in the case of 10 molecules), 200 s (in the case of 100 molecules) and 20 s (in the case of 1000 molecules). The number of lacI molecules and the association rates were varied according to the system size, see Table 4.

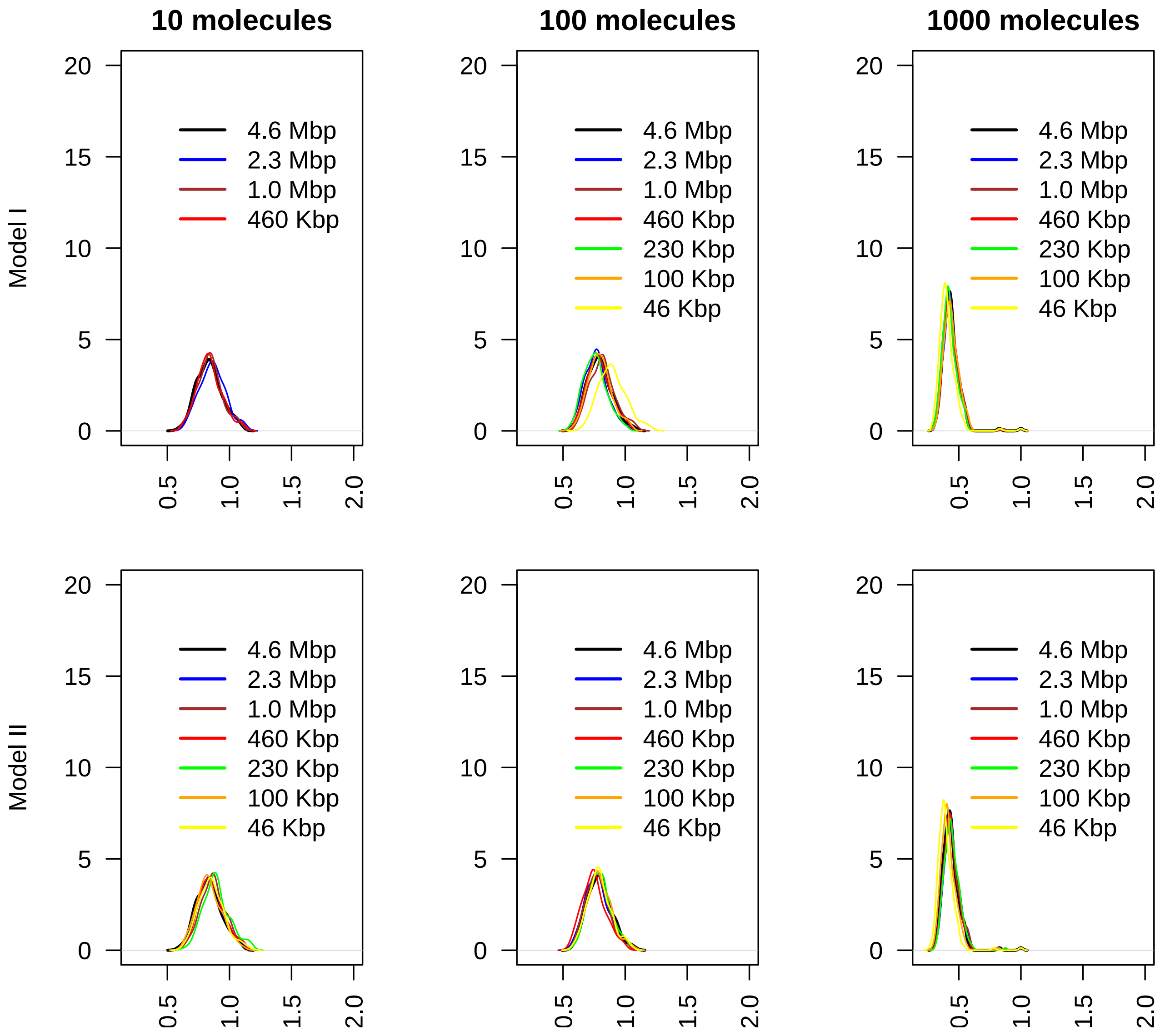

Figure 11: *The ratio between normalized affinity and normalized occupancy when the subsystems are scaled with the total affinity ratio* We consider the smallest subsequence (46 Kbp) and consider the top $\approx$ 180 sites (the binding energy is not lower than 30% compared to the strongest site). Only lacI molecules were added to the system and we ran 60 simulations for each set of parameters, each simulation was run for: 2000 s (in the case of 10 molecules), 200 s (in the case of 100 molecules) and 20 s (in the case of 1000 molecules). The number of lacI molecules and the association rates were varied according to the system size, see Table 4.

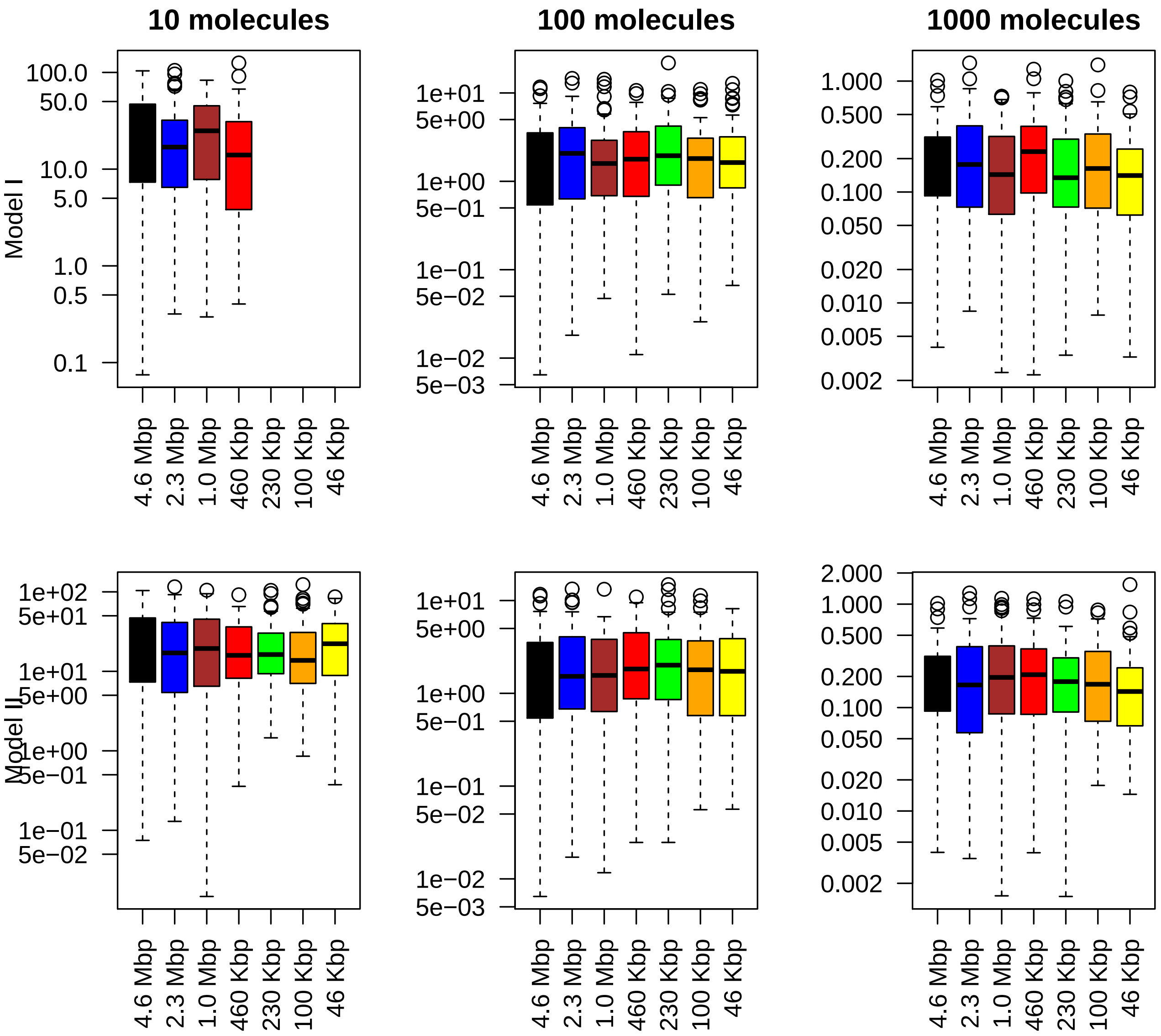

Figure 12: *The time to reach the target site when the subsystems are scaled with the total affinity ratio* We ran 60 independent simulations, only lacI molecules were added to the system and each simulation was run for: 2000 s (in the case of 10 molecules), 200 s (in the case of 100 molecules) and 20 s (in the case of 1000 molecules). The number of lacI molecules and the association rates were varied according to the system size, see Table 4.

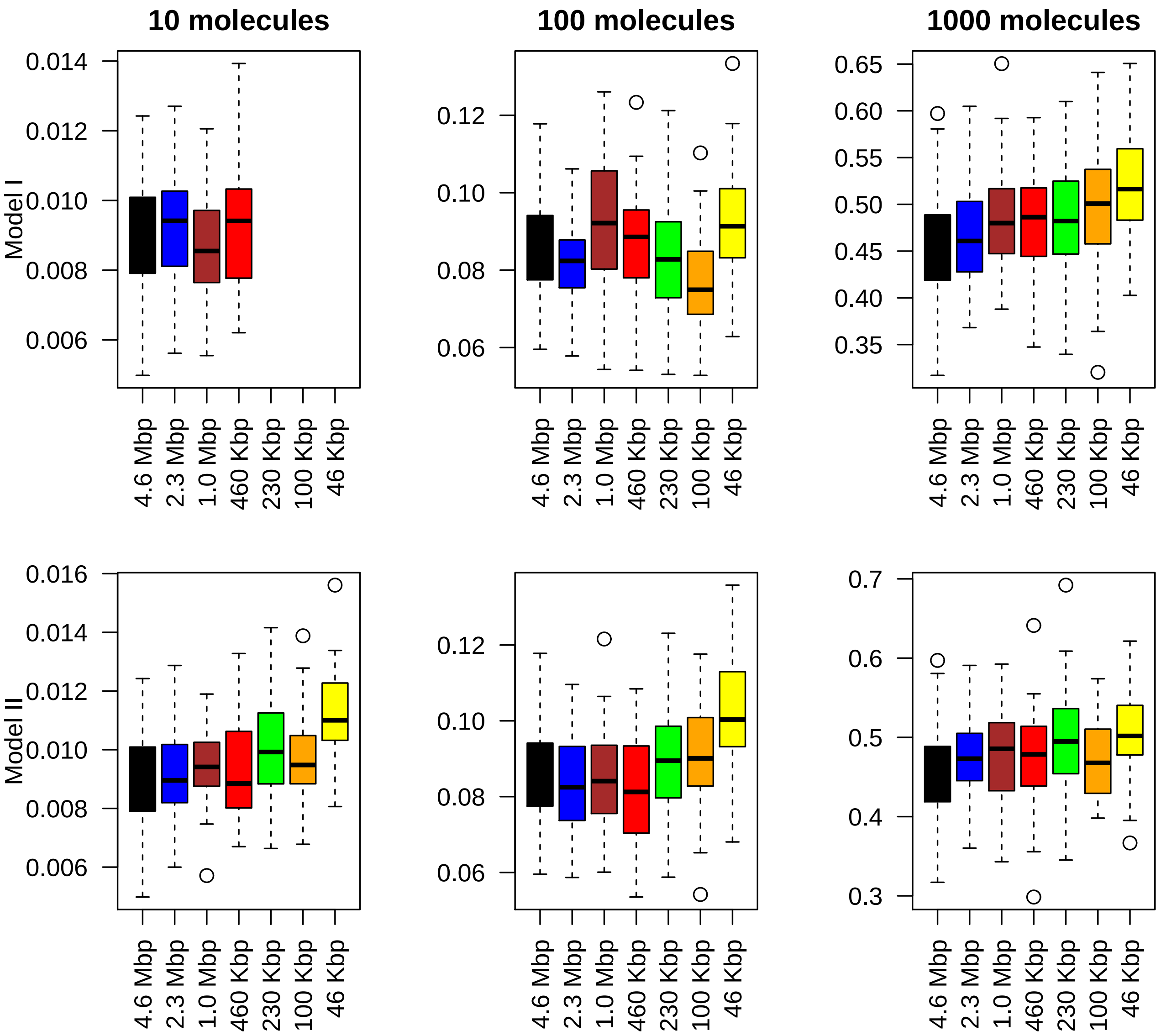

Figure 13: *The probability that the target site is occupied by a TF molecule when the subsystems are scaled with the total affinity ratio* We measure the proportion of time the O_1 target site was occupied by lacI molecules during the simulation time using 60 independent simulations. Only lacI molecules were added to the system and each simulation was run for: 2000 s (in the case of 10 molecules), 200 s (in the case of 100 molecules) and 20 s (in the case of 1000 molecules). The number of lacI molecules and the association rates were varied according to the system size, see Table 4.

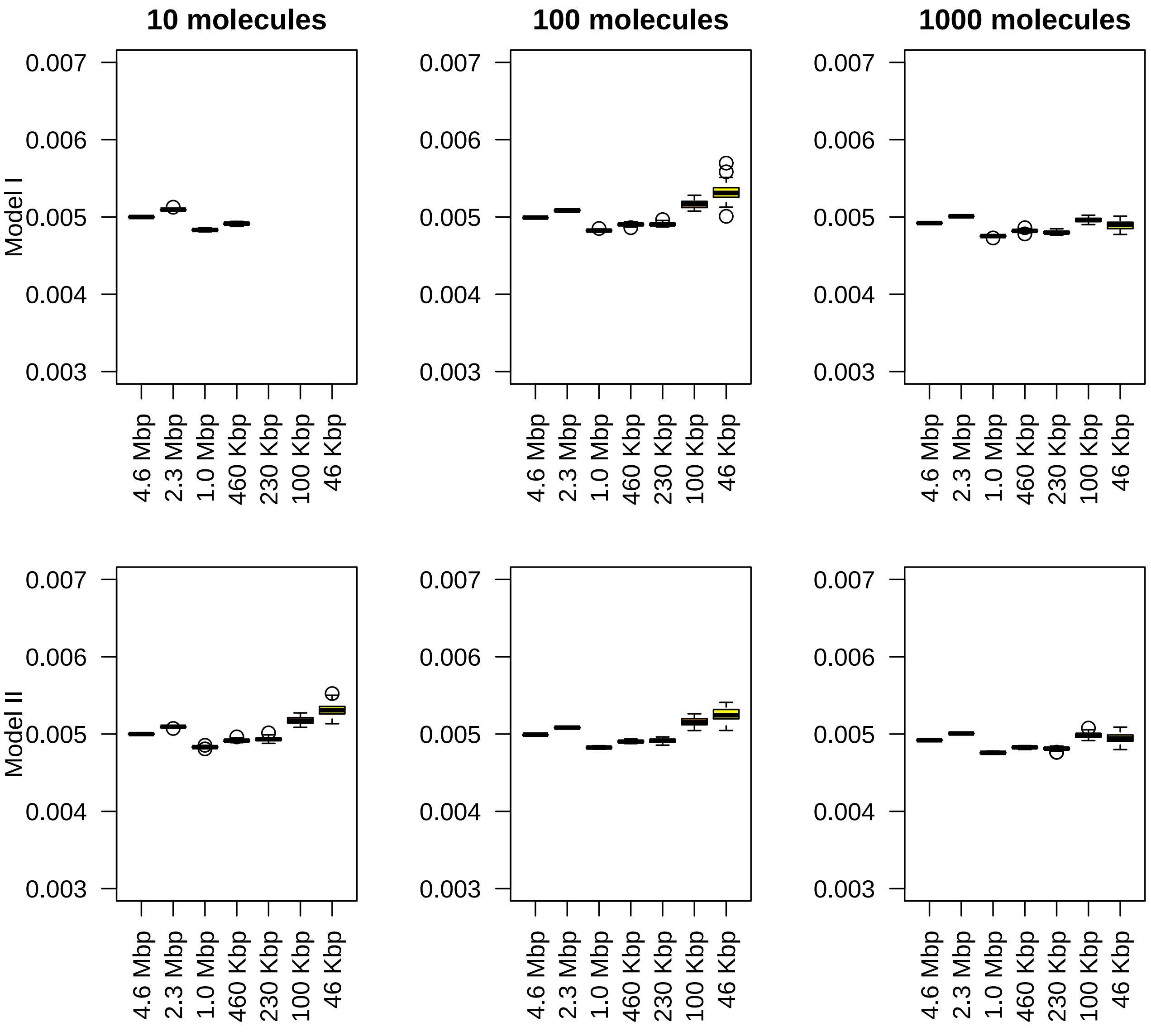

Figure 14: *The residence time per binding when the subsystems are scaled with the total affinity ratio.* We ran 60 independent simulations, only lacI molecules were added to the system and each simulation was run for: 2000 s (in the case of 10 molecules), 200 s (in the case of 100 molecules) and 20 s (in the case of 1000 molecules). The number of lacI molecules and the association rates were varied according to the system size.

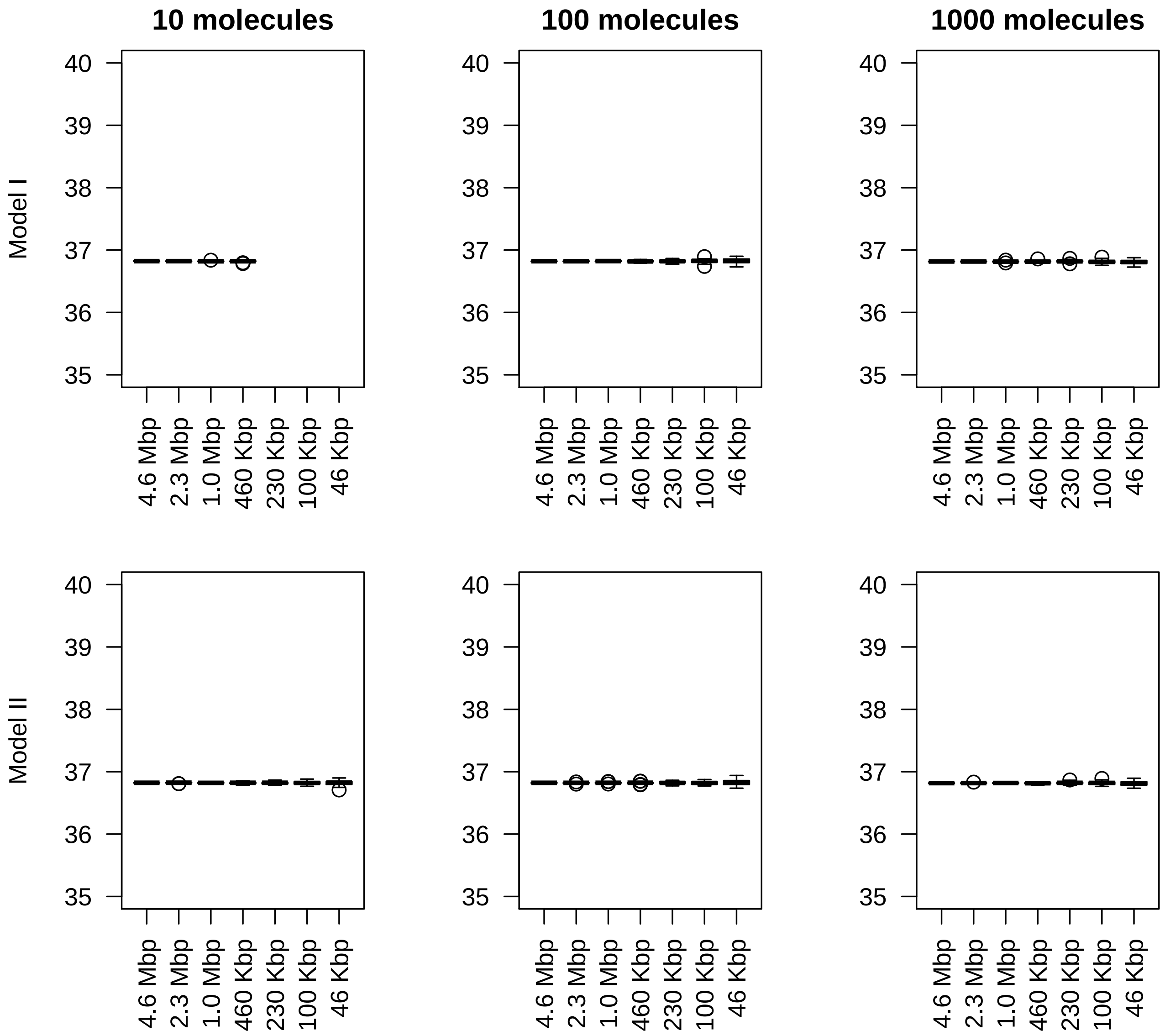

Figure 15: *The sliding length when the subsystems are scaled with the total affinity ratio.* We ran 60 independent simulations, only lacI molecules were added to the system and each simulation was run for: 2000 s (in the case of 10 molecules), 200 s (in the case of 100 molecules) and 20 s (in the case of 1000 molecules). The number of lacI molecules and the association rates were varied according to the system size.

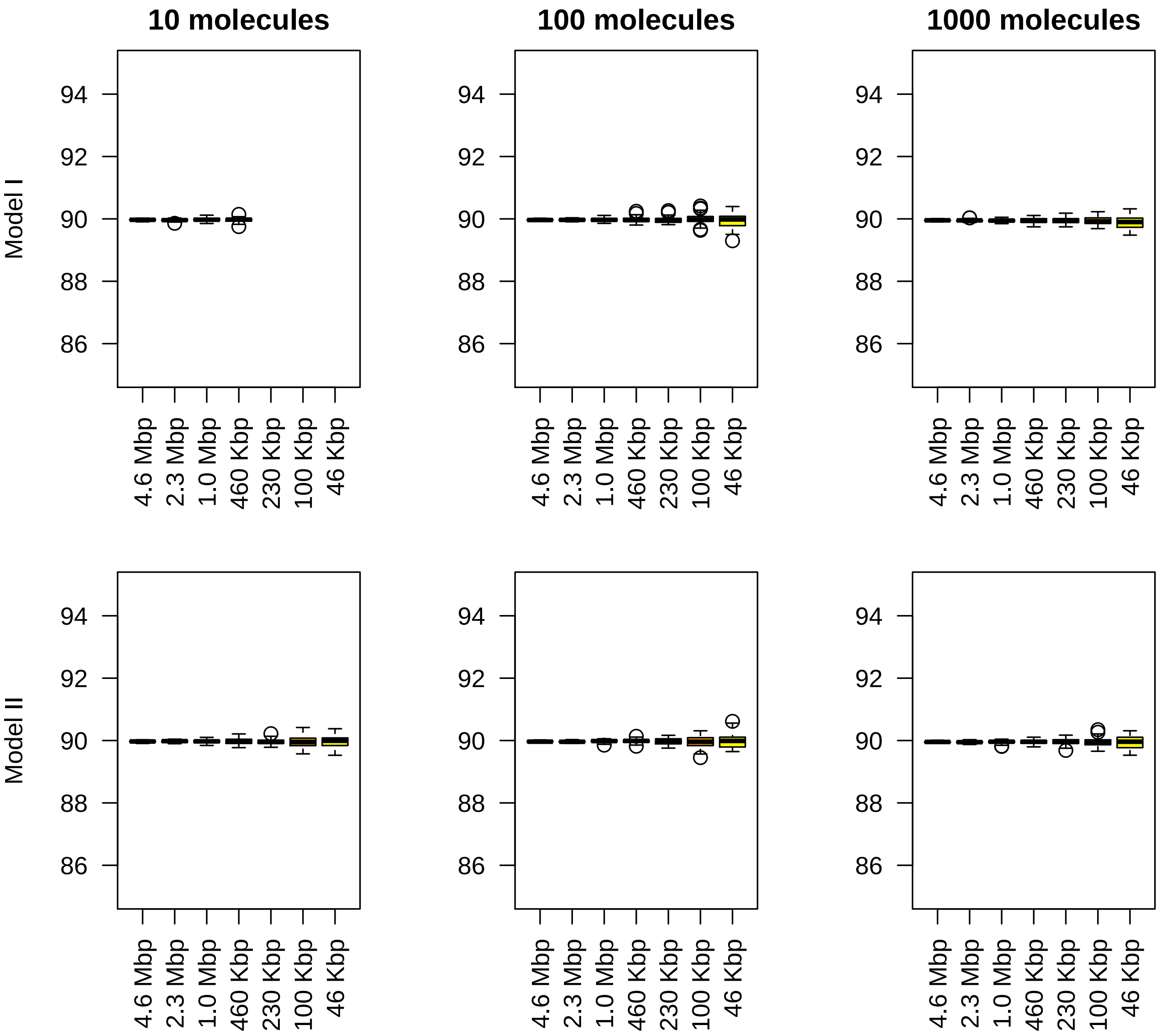

Figure 16: *The actual sliding length when the subsystems are scaled with the total affinity ratio.* We ran 60 independent simulations, only lacI molecules were added to the system and each simulation was run for: 2000 s (in the case of 10 molecules), 200 s (in the case of 100 molecules) and 20 s (in the case of 1000 molecules). The number of lacI molecules and the association rates were varied according to the system size.

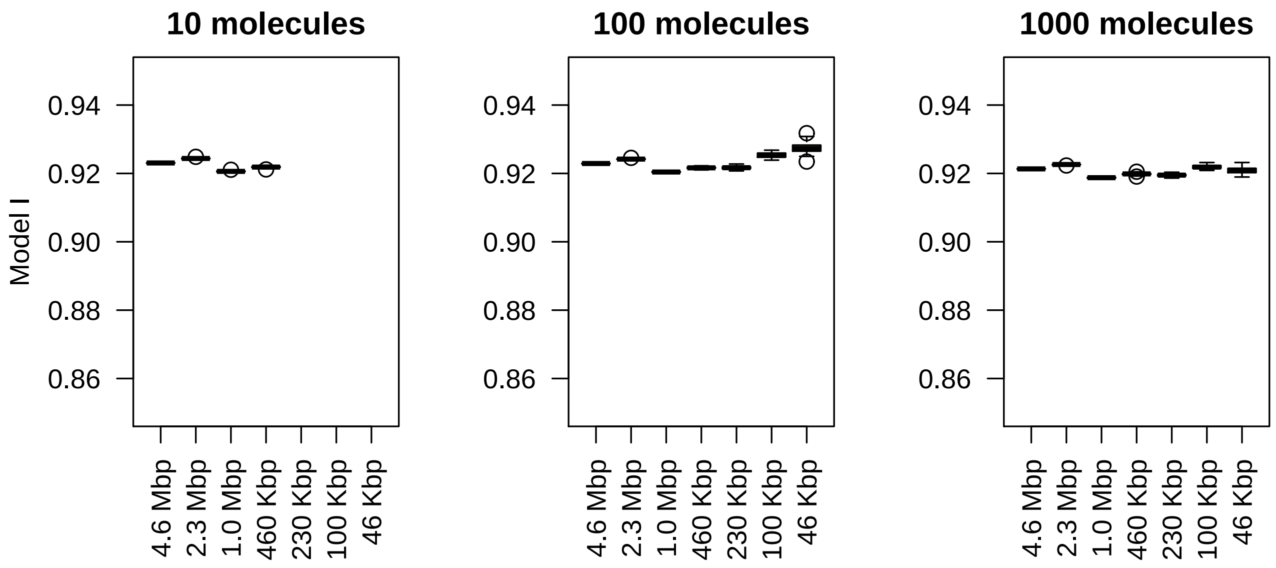

Figure 17: *The proportion of time a Tf molecule is bound to the DNA when the subsystems are scaled with the total affinity ratio.* We ran 60 independent simulations, only lacI molecules were added to the system and each simulation was run for: 2000 s (in the case of 10 molecules), 200 s (in the case of 100 molecules) and 20 s (in the case of 1000 molecules). The number of lacI molecules and the association rates were varied according to the system size. The values for Model II decrease due to the fact that we alter the parameters in order to obtain less time spent on the DNA.

References

Gerland, U., Moroz, J. D., and Hwa, T. (2002). Physical constraints and functional characteristics of transcription factor-DNA interactions. *PNAS*, 99(19):12015–12020.

Riley, M., Abe, T., Arnaud, M. B., Berlyn, M. K., Blattner, F. R., Chaudhuri, R. R., Glasner, J. D., Horiuchi, T., Keseler, I. M., Kosuge, T., Mori, H., Perna, N. T., Plunkett, G., Rudd, K. E., Serres, M. H., Thomas, G. H., Thomson, N. R., Wishart, D., and Wanner, B. L. (2006). Escherichia coli k-12: a cooperatively developed annotation snapshot - 2005. *Nucleic Acids Research*, 34(1):1–9.

Stormo, G. D. (2000). DNA binding sites: representation and discovery. *Bioinformatics*, 16(1):16–23.

Vilar, J. M. G. (2010). Accurate prediction of gene expression by integration of DNA sequence statistics with detailed modeling of transcription regulation. *Biophysical Journal*, 99:2408–2413.

Zabet, N. R. and Adryan, B. (2012). A comprehensive computational model of facilitated diffusion in prokaryotes. *Bioinformatics*, 28(11):1517–1524.
